# Genetic evidence for the origin of *Aedes aegypti*, the yellow fever mosquito, in the southwestern Indian Ocean

**DOI:** 10.1111/mec.15590

**Published:** 2020-08-30

**Authors:** John Soghigian, Andrea Gloria‐Soria, Vincent Robert, Gilbert Le Goff, Anna‐Bella Failloux, Jeffrey R. Powell

**Affiliations:** ^1^ Yale University New Haven CT USA; ^2^ Department of Entomology and Plant Pathology North Carolina State University Raleigh NC USA; ^3^ Center for Vector Biology & Zoonotic Diseases Department of Environmental Sciences The Connecticut Agricultural Experiment Station New Haven CT USA; ^4^ MIVEGEC Unit IRD Montpellier Univ. CNRS Montpellier France; ^5^ Institut Pasteur Arboviruses and Insect Vectors Paris France

**Keywords:** insects, invasive species, population genetics—empirical, systematics

## Abstract

*Aedes aegypti* is among the best‐studied mosquitoes due to its critical role as a vector of human pathogens and ease of laboratory rearing. Until now, this species was thought to have originated in continental Africa, and subsequently colonized much of the world following the establishment of global trade routes. However, populations of this mosquito on the islands in the southwestern Indian Ocean (SWIO), where the species occurs with its nearest relatives referred to as the Aegypti Group, have received little study. We re‐evaluated the evolutionary history of *Ae. aegypti* and these relatives, using three data sets: nucleotide sequence data, 18,489 SNPs and 12 microsatellites. We found that: (a) the Aegypti Group diverged 16 MYA (95% HPD: 7–28 MYA) from its nearest African/Asian ancestor; (b) SWIO populations of *Ae. aegypti* are basal to continental African populations; (c) after diverging 7 MYA (95% HPD: 4–15 MYA) from its nearest formally described relative (*Ae. mascarensis*), *Ae. aegypti* moved to continental Africa less than 85,000 years ago, where it recently (<1,000 years ago) split into two recognized subspecies *Ae. aegypti formosus* and a human commensal, *Ae. aegypti aegypti*; (d) the Madagascar samples form a clade more distant from all other *Ae. aegypti* than the named species *Ae. mascarensis*, implying that Madagascar may harbour a new cryptic species; and (e) there is evidence of introgression between *Ae. mascarensis* and *Ae. aegypti* on Réunion, and between the two subspecies elsewhere in the SWIO, a likely consequence of recent introductions of domestic *Ae. aegypti aegypti* from Asia.

## 1 INTRODUCTION

The *Aedes aegypti* mosquito is often considered to be among the most dangerous animal in the world due to its ability to transmit several arboviruses (yellow fever, dengue, chikungunya and Zika) that historically have taken a heavy toll on human health and continue to do so today (Powell, 2016). The European colonization of the New World was strongly affected by *Ae. aegypti*, and these events define the Americas today (McNeill, 2010). Beyond its direct role in disease transmission, this mosquito is easy to rear in the laboratory and thus has been a model organism for laboratory‐based disciplines such as physiology, neurobiology and development (Christophers, 1960; Clements, 1999a, 1999b, 2012). Its evolutionary genetics has been well studied for four decades (Crawford et al., 2017; Gloria‐Soria et al., 2016; Paupy, Vazeille‐Falcoz, Mousson, Rodhain, & Failloux, 2000; Rašić, Filipović, Weeks, & Hoffmann, 2014; Tabachnick & Powell, 1979), and it has been a model for invasive species biology (Braks, Honório, Lounibos, Lourenço‐de‐Oliveria, & Juliano, 2004; Brown et al., 2014).

*Aedes aegypti* is globally distributed in the tropics and subtropics. Ancestrally, there is little doubt that it occupied sub‐Saharan Africa, where populations are still found in tropical rainforests, with larvae breeding in tree holes and female adults taking bloodmeals from nonhuman mammals (Lounibos, 1981; McBride et al., 2014). As human settlements grew in Africa, populations of this mosquito evolved to become associated with human habitats, where larvae can be found in human‐generated containers and females prefer humans for bloodmeals. About 500 years ago, this human‐associated form left Africa, likely via slave trade, and first invaded the New World, then subsequently Asia and the Pacific Islands, including Australia (reviewed in Powell, Gloria‐Soria, & Kotsakiozi, 2018). These two forms, one sylvatic in continental Africa and one domestic, primarily outside Africa, have been given subspecific names: *Ae. aegypti formosus* (abbreviated Aaf) and *Ae. aegypti aegypti* (Aaa), respectively. The vast majority of work has been on these two subspecies that we here refer to as *Aedes aegypti* sensu* stricto* (s.s.), to distinguish them from the newly studied populations from the islands in the southwestern Indian Ocean, which we will refer to here as *Aedes aegypti* sensu *lato* (s.l.). We note that while the clear‐cut distinction of the subspecies designations is questionable in some instances (e.g. Powell & Tabachnick, 2013), here we use Aaf and Aaa as convenient shorthand for African native and outside Africa populations, respectively.

**FIGURE 1:**
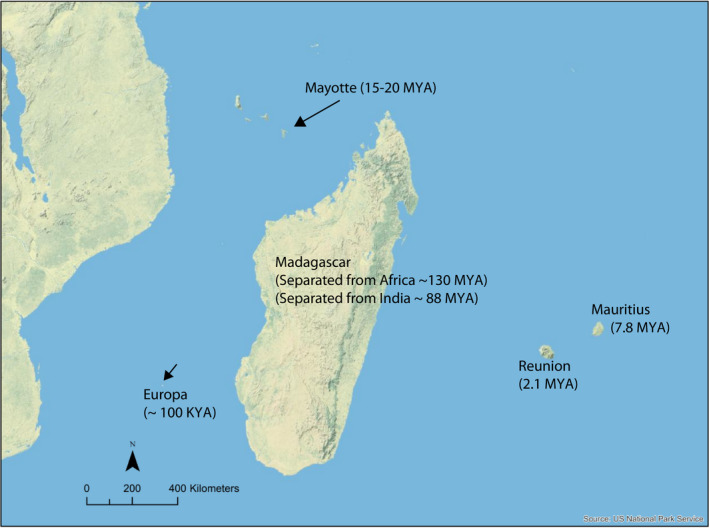
Map of the southwestern Indian Ocean, east of Africa. Estimates of island ages are from Ashwal et al. (2017), Warren et al. (2003) and Michon (2016). While *Ae. aegypti* is found on Madagascar and all islands highlighted, *Ae. mascarensis* is endemic to Mauritius, and *Ae pia* is endemic to Mayotte [Colour figure can be viewed at wileyonlinelibrary.com]

While the evolutionary history and genetic diversity of *Ae. aegypti* s.s. have received substantial study, populations of *Ae. aegypti* (and close relatives) on the islands east of Africa have received comparatively little attention (Failloux, Vazeille, & Rodhain, 2002; Kotsakiozi, Evans, et al., 2018; Vazeille et al., 2001). In this region of the southwestern Indian Ocean, *Ae. aegypti* occupies Madagascar and numerous smaller islands between Africa and Madagascar, such as Mayotte and Europa, as well as islands east of Madagascar, such as Réunion and Mauritius (Figure 1). A previous study estimated that Malagasy *Ae. aegypti* diverged from their continental African counterparts approximately 10 MYA (Fort et al., 2012).

In addition to *Ae. aegypti*, two closely related species from these islands have been formally described. These are *Ae. mascarensis*, endemic on Mauritius (MacGregor, 1924); and *Ae. pia*, endemic on Mayotte (Le Goff, Brengues, & Robert, 2013). Together with *Ae. aegypti* s.s., these three species, in the subgenus *Stegomyia,* compose the Aegypti Group (Huang, 2004). *Aedes mascarensis* and *Ae. aegypti* s.s. can mate in the laboratory forming fertile F_1_ offspring, but hybrid viability breaks down after the first generation (Hartberg & Craig, 1970). Genetic analysis of *Ae. aegypti* s.l. from Réunion has suggested the possibility of natural introgression from *Ae. mascarensis* (Kotsakiozi, Evans, et al., 2018). *Aedes pia* was described in 2013 (Le Goff et al., 2013), and is basal in phylogenetic analyses to all other members of the Aegypti Group (Soghigian, Andreadis, & Livdahl, 2017). The reproductive compatibility of *Ae. pia* with other members of the Aegypti Group is unknown.

In this paper, we push back considerably longer in time our understanding of the evolutionary history of *Ae. aegypti*, to the origin of *Ae. aegypti* s.s. on continental Africa. New data from populations on southwestern Indian Ocean islands provide evidence for novel insights.

## 2 METHODS

### 2.1 Mosquito collection and DNA extraction

*Aedes aegypti* were collected by our group, as well as collaborators from various geographic locations worldwide, particularly from the islands in the southwestern Indian Ocean (Table S1) and were processed at Yale University, New Haven, USA. DNA was extracted for all samples, other than *Ae. pia,* with the Qiagen DNeasy Blood and Tissue Kit (Qiagen) following the manufacturer's recommendations and including the optional RNase A step, and stored at −20°C until further use. *Aedes pia* is a morphologically distinct member of the subgenus *Stegomyia* and is basal to the other two members of the Aegypti species group: *Aedes aegypti* and *Aedes mascarensis* (Soghigian et al., 2017). DNA from two adult *Aedes pia* was extracted from pinned specimens, using phenol–chloroform and genotyped as described for the other specimens. We also collected adult specimens of *Culiseta melanura*, *Culiseta inornata* and *Culex erraticus* from Worcester, for use in divergence time analyses, below.

### 2.2 Estimating the evolutionary history of the Aegypti Group with nucleotide data

To estimate the evolutionary relationships and date the divergence times in the Aegypti Group, we generated new sequence data for *Ae. aegypti* from Madagascar and other species (see below), and we used previously published sequence data from Soghigian et al. (2017) for mosquitoes from the genus *Aedes*, subgenus *Stegomyia*. The subset of Soghigian et al. (2017) contained nucleotide data from *Stegomyia* for 25 species from 12 species groups (Table S2), including five of the ten species groups found in Africa (Huang, 2004). Soghigian et al. (2017) analysed an alignment of sequence data from 200 *Aedes* mosquitoes, generated from both sequencing of specimens and data mining of GenBank, to study the evolution of larval habitat specialization in *Aedes*; this work did not focus on *Stegomyia* specifically, but contained numerous species from this subgenus. From this original data set, we also used outgroups from *Psorophora* and *Culex* (supplemented by additional outgroups sequenced for this study; see below) for which fossil calibrations are known. We used nucleotide sequence data for estimating the evolutionary relationships of *Stegomyia*, rather than SNP data detailed elsewhere, as the SNP chip data captured from samples more distant from *Ae. aegypti s.s*. is limited (see supplemental information for missing data discussion) and because fossil calibrations are not available for the Aegypti Group but are for *Aedes* and other Culicidae (see below and supplemental information). The alignment derived from Soghigian et al. (2017) included partitions for seven markers: 18S, 28S, enolase, arginine kinase, cytochrome oxidase I and II, and ITS2. This alignment was merged with additional sequence data for *Ae. aegypti* from Madagascar and species for which additional fossil calibrations were available (see below and supplemental information), generated following sequencing protocols detailed more thoroughly in Soghigian et al. (2017). In brief, the aforementioned markers were amplified using previously published primers (Cook, Diallo, Sall, Cooper, & Holmes, 2005; Folmer, Black, Hoeh, Lutz, & Vrijenhoek, 1994; Reidenbach et al., 2009; Soghigian et al., 2017; Wesson, Porter, & Collins, 1992), given in Table S3, then sequenced in both directions by Macrogen (Macrogen Boston USA). Using Geneious version 9 (Drummond et al., 2012) and the MAFFT version 7 (Katoh, Misawa, Kuma, & Miyata, 2002) alignment software, new sequences from *Ae. aegypti* from Madagascar, *Cs. melanura*, *Cs. inornata* and *Cx. erraticus*, were added to the existing alignment of each marker from Soghigian et al. (2017), such that the new alignment was 6,471 base pairs from 33 taxa. Hereafter, we will refer to this as our nucleotide data set.

We estimated the evolutionary history of the Aegypti Group, and the rest of the subgenus *Stegomyia*, with the nucleotide data set in IQ‐Tree (Hoang, Chernomor, von Haeseler, Minh, & Vinh, 2018; Kalyaanamoorthy, Minh, Wong, von Haeseler, & Jermiin, 2017; Nguyen, Schmidt, von Haeseler, & Minh, 2015). We partitioned our data set according to marker, and by codon position for protein‐coding genes. We then allowed IQ‐Tree to choose the best‐fitting substitution model per partition, and we assessed clade support using ultrafast bootstrap values. In order to determine whether our mitochondrial markers (cytochrome oxidase I and II) had concordant topologies with our nuclear data (18S, 28S, ITS2, arginine kinase and enolase), we repeated this analysis with only the mitochondrial partitions and with only nuclear partitions, and compared subsequent results with our topology from all seven markers, as well as compared our results with the phylogeny of the Aegypti Group obtained from SNPs (see below). We included several *Stegomyia* species missing loci (see Table S2) in order to increase the breadth of our taxonomic sampling of the subgenus. Although missing data are generally thought not to influence phylogenetic results in terms of topology or support values (Pyron, Burbrink, & Wiens, 2013; Roure, Baurain, & Philippe, 2013; Wiens & Morrill, 2011; Wiens & Tiu, 2012), we evaluated whether the degree of missing data at some markers might influence relative branch lengths, as these branch lengths would be essential for accurate divergence time estimates. Following Pyron et al. (2013), we used Pearson's correlation to test whether the terminal branch length from the ML analysis of the full nucleotide data set was correlated for (a) the number of loci present in the analysis, and (b) the proportion of nucleotides in the alignment.

Next, we used BEAST2 (Bouckaert et al., 2014) to estimate divergence times among members of the Aegypti Group. We used bModelTest (Bouckaert & Drummond, 2017) to estimate substitution models for each partition for ribosomal RNA genes, and by codon position for protein‐coding genes. We used a birth–death tree process and a relaxed clock model following recommendations for BEAST2 divergence time analyses (Barido‐Sottani et al., 2018). We placed four fossil calibrations, including one fossil from the genus *Aedes* and three outside of the genus, with uniform priors set to minimum ages based on minimum fossil age, and maximum ages set to previous estimates from Reidenbach et al. (2009; see Table S16 for fossils, constrains and explanation). We ran the BEAST2 analysis for 150,000,000 generations on CIPRES (Miller, Pfeiffer, & Schwartz, 2010), after which we evaluated logs to ensure convergence of parameter estimates and subsequently generated a maximum clade credibility tree with TreeAnnotator from BEAST2 with common ancestor heights and 95% highest posterior density (HPD) intervals. We ran two additional chains to confirm consistent estimates of divergence time across chains.

### 2.3 SNP genotyping, calling and filtering

For fine‐scale population structure and admixture analysis, we generated a SNP data set with populations of *Ae. aegypti* from throughout its global distribution, and from other members of the Aegypti Group, with a focus on the southwestern Indian Ocean. Approximately 200 ng of genomic DNA per individual mosquito was genotyped with the *Ae. aegypti* Axiom_aegypti1 SNP chip (Life Technologies Corporation CAT#550481; Evans et al., 2015), at the University of North Carolina Functional Genomics Core, Chapel Hill. Additionally, we used previously described samples from Gloria‐Soria et al. (2018) and Kotsakiozi, Evans, et al. (2018) from several populations of *Aedes aegypti* collected worldwide, as well as for *Ae. mascarensis* from the island of Mauritius (Table S1). For population analyses, we classified populations into five groups corresponding to currently accepted species and subspecies designations (e.g. we divided *Aedes aegypti* s.s. into typically recognized groups of *Ae. aegypti aegypti* and *Ae. aegypti formosus*), and a group for "island" populations of *Ae. aegypti* originating from Europa, Madagascar, Mayotte and Réunion.

Genotype calls were made using Affymetrix Axiom Analysis Suite 3.1 (Life Technologies–Thermo Fisher Scientific), for both newly genotyped samples and previously studied populations. This integrated software suite calls genotypes from probe florescence values, excluding probes (and thus SNPs) whose fluorescence deviates from expectations, as might result from probe misbinding. For probe quality control and SNP calling, we used default software settings, save for two exceptions: first, in order to include SNPs called from *Ae. pia*, we overrode default software settings on allowable missing data for a given sample (this applied only to the two *Ae. pia* samples; see supplemental materials for a discussion on missing data); and second, we utilized the OTV Caller plugin for Axiom Analysis Suite to exclude genotype calls from SNPs that might contain off‐target variants, such as those that could occur due to insertions, deletions or mutations at probe sites. Finally, we exported all polymorphic SNPs for downstream analyses.

Resulting polymorphic SNPs were then filtered using Plink version 1.9 (Chang et al., 2015). We removed SNPs that Evans et al. (2015) found not to follow Mendelian inheritance patterns. We also removed variants present missing in more than 25% of individuals (flag ‐‐geno 0.25), alleles with a minor allele frequency lower than 0.01 (‐‐maf 0.01) and linked loci within a 75‐kb window that exceeded a variance inflation factor of 2 (‐‐indep 75k 1 2). This window size was chosen as it exceeded the linkage decay previously reported in *Ae. aegypti* (Matthews et al., 2018). We refer to this data set throughout the text as the SNP data set.

### 2.4 Microsatellite genotyping

For complementary estimations of population history and demographic parameters, we generated a microsatellite data set comprised of samples from Europa Island (hereafter Europa) and Africa. Microsatellite genotyping was performed as described previously (Gloria‐Soria et al., 2016). We used previously published microsatellite calls (Brown et al., 2011; Gloria‐Soria et al., 2016), available on VectorBase (Giraldo‐Calderón et al., 2015), for all but populations from Burkina Faso and Europa, for which data were generated in this study.

### 2.5 Genetic diversity and population structure with the SNP data set

We used our population‐level SNP data set to infer genetic diversity and population structure of *Ae. aegypti* in the southwestern Indian Ocean. Unless otherwise described, all analyses were performed in R version 3.5 (R Core Team, 2018), and maps were generated using Google Maps, or PaleoMap PaleoAtlas for GPlates (available from http://www.gplates.org/). We used a nested analysis of molecular variance (AMOVA) from the package poppr (Kamvar, Tabima, & Grünwald, 2014), defining nested strata according to (a) species/subspecies and (b) population (see Table S1) to assess whether regions and populations were significantly different from one another. We also calculated pairwise *F*_st_ values with the R package StAMPP (Pembleton, Cogan, & Forster, 2013) for subspecies and population pairs (see Table S1 for populations and subspecies), regardless of the sample size of each population, as *F*_st_ can be reliably estimated from as few as two individuals, so long as there are many thousands of markers (Nazareno, Bemmels, Dick, & Lohmann, 2017; Patterson, Price, & Reich, 2006). Next, we used sparse non‐negative matrix factorization (hereafter SNMF) as implemented in the package LEA (Frichot & François, 2015) to analyse population structure. We used the minimal cross‐entropy criterion to determine the optimal number of ancestral populations (*K* values), and also visualized alternative *K* values based on biologically reasonable ancestral clusters due to similarity in *K* values past *K* = 4. We also assessed whether the removal of the genetic cluster associated with *Aedes pia* would provide qualitatively different results from the inclusion of this outgroup species. Additionally, we performed principal component analysis to evaluate genetic structure of populations and species as implemented in the R package adegenet (Jombart & Ahmed, 2011).

### 2.6 Phylogenetic analysis with the SNP data set

We evaluated the evolutionary relationships among populations of *Ae. aegypti*, as well as other members of the Aegypti Group, using maximum‐likelihood inference on the population‐level SNP data set with ascertainment bias correction for invariant sites, as implemented in IQ‐Tree (Hoang et al., 2018; Kalyaanamoorthy et al., 2017; Nguyen et al., 2015). We allowed IQ‐Tree to select the best‐fitting substitution model, and we assessed clade support using ultrafast bootstrap values. We rooted the resulting topology on the branch leading to *Aedes pia* based on our BEAST2 analysis (above), which was consistent with previous results in Soghigian et al. (2017) that suggested *Aedes pia* was sister to *Aedes mascarensis* + *Aedes aegypti*. This choice was further supported by all other analyses that suggested *Aedes pia* was more distant from *Aedes aegypti* than *Aedes mascarensis* (or any other population), for example relative missing data (see online supplemental information) and population clustering results.

### 2.7 Admixture analysis with the SNP data set

Because our initial analysis of population structure supported a scenario of potential admixture, we used the F3 test (Reich, Thangaraj, Patterson, Price, & Singh, 2009) implemented in Treemix (Pickrell & Pritchard, 2012) to evaluate whether there was significant evidence of admixture among populations in our SNP data set. We compared population triplets, considering whether there was significant non‐tree‐like behaviour for all sets of populations, where negative *Z* scores were indicative of admixture between two parent populations to produce the third sibling population. *p*‐values were calculated from these *Z* scores with false discovery rate correction for multiple comparisons with the R function p.adjust (R Core Team, 2018).

To ensure that specific characteristics of our data set in terms of missing data and uneven sampling did not bias our admixture analysis, we estimated two additional F3 statistics per population triplet and compared them with our complete data set. First, to evaluate whether missing data influenced our Treemix results, we refiltered our data set as above, but with the flag –geno 0 in Plink, thus requiring a SNP position be present in all samples to be included. We then used this data set in Treemix, as above, to estimate F3 statistics and *p*‐values. Next, we evaluated whether the uneven sample size of some populations (e.g. *Aedes mascarensis* with four individuals relative to most populations with >11) could bias estimates of allelic variation between populations, as Treemix uses allele frequency estimates to infer admixture through the F3 test. Using the R function dplyr, we resampled, without replacement, each *Ae. aegypti* population to four individuals. For each resampling, we conducted F3 tests as implemented in Treemix as we describe above. We resampled our populations 100 times, ran Treemix on each resampled data set and then summarized the resampled mean estimates of the F3 statistic with parametric 95% confidence intervals. This summary method, rather than *p*‐values, was chosen due to the large number of three‐population comparisons (~204,000) during resampling.

### 2.8 Estimates of demographic parameters and population history with microsatellites

We complemented our inferences on population history using microsatellite markers to further investigate the divergence events that involved African *Ae. aegypti s.s*. and their relatives on Europa. Particularly, we were interested in those events that occurred more recently in evolutionary history and for which nuclear and mitochondrial genes did not resolve the divergence times or directionality. Furthermore, *Aedes aegypti* SNP chip only assays two alleles at each locus and was designed to deliberately emphasize population‐level differences among *Ae. aegypti aegypti*, possibly neglecting informative variation in African *Ae. aegypti*. Based on the admixture detected in other island populations (see below), we focused this analysis on comparisons between the Europa population, and populations from East and West Africa. Allele numbers, percentage of missing data, percentage of polymorphic loci, average observed (Ho) and expected (uHe) heterozygosities, and Fis values were estimated in GenAlEx (Peakall & Smouse, 2012). All microsatellite loci were analysed for within‐population deviations from Hardy–Weinberg equilibrium (HWE) in Genodive 2.0b.27 (Meirmans & Tienderen, 2004) with the Heterozygosity‐based (Nei) test and 10,000 permutations. A Bonferroni correction was applied to the resulting matrices of HWE to correct for multiple testing. Next, we inferred population history and demographic patterns using approximate Bayesian computation methods, ABC (Beaumont, Zhang, & Balding, 2002) as implemented in DIYABC v.2.0.4 (Cornuet et al., 2014), estimating 10 generations per year and a mutation rate ranging from 9 × 10^−6^ to 1 × 10^–5^, consistent with previous publications (Gloria‐Soria et al., 2016) and rates reported in the literature for other Diptera species (Pfeiler, Flores‐López, Mada‐Vélez, Escalante‐Verdugo, & Markow, 2013; Schug et al., 1998). ABC allowed us to explicitly contrast different hypotheses on the population history of these species, namely whether Europa diverged prior to East and West Africa, or after East or West Africa.

The ABC analysis was performed as five independent runs on microsatellite data sets, with each run containing 24 individuals belonging to populations of *Ae. aegypti* in West Africa (WA), East Africa (EA) and Europa. The data sets were from: (1) La Lope Forest (Gabon—WA) and Lunyo (Uganda—EA); (2) Ouagadougou (Burkina Faso—WA) and Kisumu (Kenya—EA); and (3–5) independent combinations of randomly drawn individuals from WA (Ngari—SE, Yaoundé—CM, La Lope Forest—GB, Ouagadougou—BF and Bijagos—GB) and EA populations (Lunyo—UG, Bundibugyo—UG, Kichwamba—UG and Kisumu—KE). We tested three scenarios (Figure S3) to determine the history of *Ae. aegypti* in continental Africa relative to Europa. Scenario 1: Europa populations gave rise to African populations; Scenario 2: WA gave rise to both Europa and EA; and Scenario 3: EA gave rise to both Europa and WA populations. Priors used for on the analysis are shown in Tables S14 and S15.

## 3 RESULTS

**FIGURE 2:**
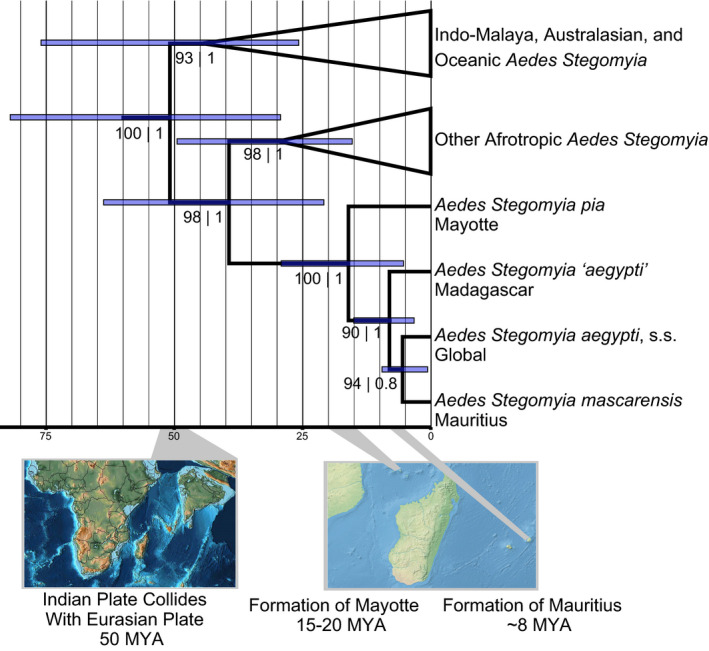
Divergence times in the Aegypti Group correlate with island formations for *Aedes pia* and *Aedes mascarensis*. Divergence times shown here based on the relaxed clock analysis in BEAST2 for members of the *Stegomyia* subgenus *Aedes*. Relationships among clades in the subgenus *Stegomyia* show here are concordant with the maximum‐likelihood analyses performed, as well (see Figure S1). The first support value indicates is the ultrafast bootstrap support value from IQ‐Tree, while the second is the posterior probability from BEAST2 [Colour figure can be viewed at wileyonlinelibrary.com]

We estimated the evolutionary history and divergence times of the members of the Aegypti Group in the southwestern Indian Ocean (Figure 2; Figures S1 and S2) with nucleotide sequence data from seven markers. Our maximum‐likelihood phylogenetic analyses on the full nucleotide data set, the mitochondrial‐only data set and the nuclear‐only data set differed in clade support values but overall recovered concordant topologies for the major lineages of *Stegomyia*, including monophyly of the Aegypti Group (Figure S1). Moreover, there was no correlation between missing data in the alignment and terminal branch length (for the number of loci in the alignment per species, *t*_31_ = 0.12, *p*‐value >0.9; and for the proportion of missing data in the alignment per species, *t*_31_ = −0.6, *p*‐value >0.5). As such, we performed divergence time estimation on the combined nuclear and mitochondrial nucleotide data set. The topology recovered from our divergence time estimate was similar to the likelihood analyses; thus, we report only the results from the divergence time analysis here (but see Figure S1).

Although sparsely sampled outside the Aegypti Group, these results (Figure 2; Figures S1 and S2) indicate a common ancestry for *Stegomyia* 52 MYA (95% HPD: 29–84 MYA) and African Stegomyia 39 MYA (95% HPD: 21–64 MYA). Our results support the monophyly of the Aegypti Group, and its affinity to other Afrotropic *Stegomyia* (Figure 2; Figure S1 and S2). Our estimates suggest a common ancestor of the Aegypti Group in the southwestern Indian Ocean 16 MYA (95% HPD: 7–28 MYA). Subsequent divergence times in the Aegypti Group suggest a divergence 7 MYA (95% HPD: 4–15 MYA) between *Ae. aegypti* on Madagascar, *Ae. mascarensis* and *Ae. aegypti* s.s., consistent with Fort et al. (2012). Of the three named species, *Ae. pia* is clearly the most distantly related and thus serves as an outgroup in later analyses.

**FIGURE 3:**
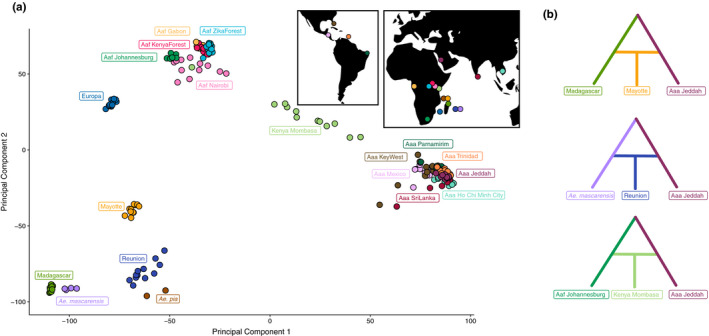
Genetic diversity in the Aegypti Group. (a) A PCA demonstrating that island populations cluster separately from the recognized subspecies *Aedes aegypti aegypti* (Aaa) and *Aedes aegypti formosus* (Aaf). (b) A visual depiction of TreeMix results demonstrating the most likely admixture scenarios involving the three admixed populations of Mayotte, Réunion and Mombasa in Kenya. In all three cases, the most likely scenario had one parental "donor" from Jeddah, although other Asian populations are possible, as well; see Sections 3 and 4 for additional details

In order to describe within‐ and between‐species genetic variation in the Aegypti Group, we genotyped individuals from global populations of *Ae. aegypti* s.s. (Table S1), including samples from three islands and Madagascar in the southwestern Indian Ocean, and two species closely related to *Ae. aegypti* (*Ae. pia* and *Ae. mascarensis*), with the *Ae. aegypti* SNP chip (Evans et al., 2015). Following filtering (see Section 2), we retained 18,489 SNPs for subsequent analyses with an overall genotyping rate of 0.95. Among subspecies and populations, missing data were highest in *Ae. pia*, and lowest in populations of *Ae. aegypti* (see our discussion of missing data in the online supplemental materials). An analysis of molecular variation on our SNP data found significant genetic differentiation among subspecies Aaf and Aaa, island populations and species in the Aegypti Group (Table S4). Pairwise *F*_st_ values among these groups indicated that *Ae. pia* was the most distinct, with island populations having lower mean *F*_st_ values to *Ae. mascarensis* than to Aaa and Aaf (Tables S4–S6). These results are consistent with the PCA of the SNP data, which supports the uniqueness of these island populations as distinct from all other *Ae. aegypti* s.s. (Figure 3a). Population structure as assessed by the STRUCTURE‐like SNMF was also consistent with the PCA and phylogenetic results (see Figure S4 for SNMF). At the optimal *K* determined by cross‐entropy, *K* = 3, island populations had highest identity with the genetic clusters associated with *Ae. aegypti* from Madagascar, *Ae. mascarensis* and *Ae. pia*, than with the clusters associated with Aaa or Aaf. However, at this and higher *K* values (Figure S4), individuals from island populations frequently contained a minority of ancestry to ancestral populations associated with Aaa or Aaf, potentially indicating admixture.

**FIGURE 4:**
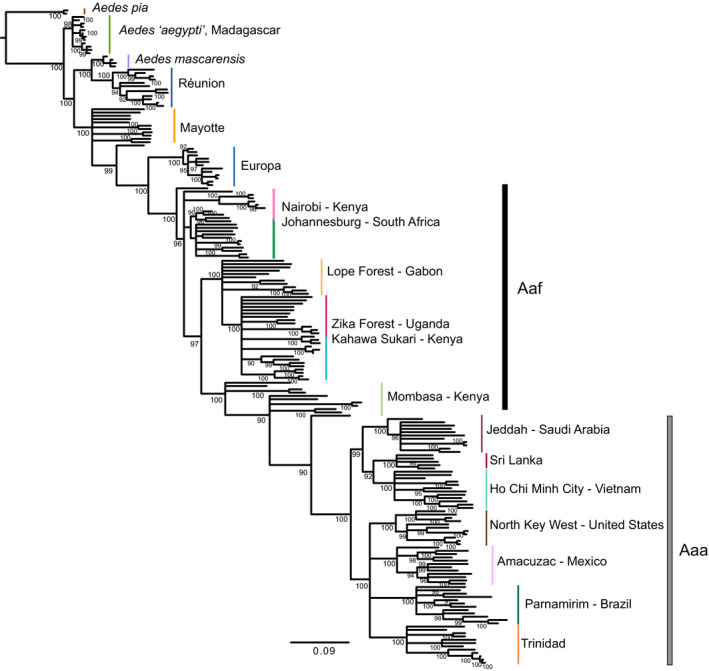
The maximum‐likelihood phylogeny estimated from Aegypti Group SNP data demonstrates the deep divergence of *Aedes aegypti* from Madagascar from populations of *Aedes aegypti* s.s. Narrow coloured vertical bars reflect species or population of origin. Black and grey bars reflect populations of *Aedes aegypti* s.s. assigned to one of the two subspecies *Aedes aegypti formosus* (Aaf) or *Aedes aegypti aegypti* (Aaa). The phylogeny was estimated in IQ‐Tree and was rooted on the branch leading to *Aedes pia*. Support values are ultrafast bootstraps estimated in IQ‐Tree, and branches with support values below 90 have been collapsed. Scale is in substitutions per site

A maximum‐likelihood phylogenetic analysis of the SNP data indicated that all southwestern Indian Ocean populations are basal to *Ae. aegypti* s.s. (Figure 4). Consistent with the maximum‐likelihood‐based phylogenetic and Bayesian divergence time analyses above, *Ae. aegypti* from Madagascar resolved as basal to all other *Ae. aegypti* populations and *Ae. mascarensis*. The Réunion population of *Ae. aegypti* resolved as sister to *Ae. mascarensis*, while Mayotte and Europa occupied intermediate regions of the phylogenetic tree. *Ae. aegypti* s.s. formed a monophyletic group that includes all sampled Aaf populations from continental Africa and all Aaa populations from outside Africa.

Due to the aforementioned analyses indicating evidence of admixture in the southwestern Indian Ocean, we utilized F3 tests implemented in Treemix to formally test for potential admixture, with three separate analyses: the primary full SNP data set (18,489 SNPs), a data set with no missing data derived from this data set (5,866 SNPs) and a data set where populations of *Ae. aegypti* were subsampled to four individuals (and resampled 100 times). Since the results of our primary SNP data set achieved qualitatively the same conclusion to that of the other two data sets investigated, and because of the increased power of the larger sample size in terms of SNPs and number of individuals per population, we report the specific results from the primary data set here (but see supplemental tables). Tests of admixture (Figure 3b; Tables S7–S9) found strong evidence that three populations were the result of admixture: Mayotte, with evidence of admixture between *Aedes aegypti* from Madagascar and a population of Aaa, with Madagascar + Jeddah assigned the lowest *Z* score (*Z* = −21.34, *p* = 5.58E‐101); Réunion, with evidence of admixture between *Ae. mascarensis* and a population of Aaa, with *Ae. mascarensis* + Jeddah again assigned the lowest Z score (*Z* = −10.09, *p* = 6.36E‐24); and Mombasa, the likely result of admixture between a population of Aaf and a population of Aaa, with Johannesburg + Jeddah assigned the lowest *Z* score (*Z* = −41.77, *p* < 2E‐200). Both the filtered‐for‐completeness data set and the resampled subset of four *Ae. aegypti* individuals per population found the same likely admixed populations and same originating parent populations having the lowest F3 statistics as in the primary analysis. However, the F3 statistics estimated were lower, concordant with the reduced power from fewer SNPs and/or fewer individuals per population from which to estimate allele frequencies.

Although Jeddah was a common contributor in the lowest F3 scenario across all analyses, all Asian populations tested were suggested as putative ancestors of these admixed populations (Tables S7, S8 and S19). As such, any Asian population in our analysis could also have been a source of admixture in this region. Unlike other island populations, we found no evidence of admixture within mosquito samples from Madagascar or Europa.

Next, we used microsatellite markers and approximate Bayesian computation (ABC) to confirm that the population of Europa, sister to *Ae. aegypti* s.s. in our maximum‐likelihood analysis, diverged prior to continental *Aedes aegypti* diversifying within Africa, rather than representing a re‐introduction from the continent. We used representative populations from East and West Africa to explicitly model whether populations in Europa diverged prior to, or after, the divergence of East and West African populations of *Aedes aegypti* on continental Africa (Kotsakiozi, Evans, et al., 2018). As our main focus was on populations in the southwestern Indian Ocean, and because the population structure of African *Ae. aegypti* has been recently reported from these microsatellites (Gloria‐Soria et al., 2016), we report detailed population statistics from microsatellites in our supplement but summarize our descriptive statistics on the microsatellite data set briefly here. The main microsatellite data set used for this analysis included genotypes from 12 loci with 159 total alleles (Table S10). This data set contained individuals from six populations from West Africa and four from East Africa, plus the Europa population, and had 1.02% missing data (Table S11). Population genetic statistics for individual locations are reported in Table S12. The average observed heterozygosity (Ho) across the data set was 0.567, with Ho = 0.458 in Europa, Ho = 0.579 in West Africa and Ho = 0.577 in East Africa; see Table S12. A total of 14 out of 143 (9.7%) population‐by‐locus comparisons deviate significantly from Hardy–Weinberg expectations, after sequential Bonferroni correction (Table S13). The ABC analysis on the African and Europa Island samples of *Ae. aegypti* supports a scenario where the colonization of Africa occurred from the Indian Ocean (represented by Europa) less than 85,000 years ago (Mean: 36,500 YA, 95% CI across runs: 9,780–84,300 YA; see Tables S14 and S15), with a predicted split between West and East Africa no older than 50,000 years ago (mean: 21,000 YA, 95% CI across runs: 7,810–49,400 YA; see Tables S14 and S15), assuming an average of 10 generations per year (Scenario 1: *p* = .8864 and *p* = .8481 in Tables S14 an S14; Figure S3). Alternative scenarios were poorly supported by the analysis (*p* < .11; Tables S14 and S15; Figure S3). The estimated mutation rate under the best‐fit scenario was ~9.5 × 10^−6^, and falls within the range of microsatellite mutation rates estimated for other Diptera (Pfeiler et al., 2013; Schug et al., 1998).

## 4 DISCUSSION

### 4.1 The Origin of the Aegypti Group and *Ae. aegypti* s.s

Research in the evolutionary history of this medically important group of mosquitoes has long focused on continental Africa and the invasive range throughout the subtropics and tropics, where most epidemics of the diseases transmitted by *Ae. aegypti* have occurred. But to fully understand the history of this mosquito, we need to consider its relationship to other members of the subgenus *Stegomyia* and, in particular, other members of the Aegypti Group. Our analysis (Figure 2; Figures S1 and S2) found two well‐supported clades of *Stegomyia*, corresponding to an Afrotropic lineage and an Indo‐Malaya/Oceanic lineage (following the biogeographic regions proposed in Olson et al., 2001), with a last common ancestor approximately 50 MYA, although the large HPD intervals on our divergence estimates indicate a high degree of uncertainty on this estimate. The estimated age is consistent with the time period in which the Asian and Indian plates collided. Given the distribution of many *Stegomyia* species around the Indian Ocean, it is tempting to associate this geologic event with the subsequent distribution of *Stegomyia*, but our limited sampling and large intervals around divergence time estimates preclude strong conclusions on the biogeographic origin of the subgenus as a whole. Our reliance on uniform prior densities for fossil calibrations, due to the relatively poor record of fossil mosquitoes, uncertainty regarding the age of mosquito clades and the limited sequence data at hand to place additional fossils, likely resulted in large intervals around our divergence time estimates. Nevertheless, our analyses indicate that one lineage of Afrotropic *Stegomyia* diversified in Africa (leading to *Ae. africanus*, *Ae. simpsoni*, and others; see Figure 2). Another lineage, which entered the islands of the southwestern Indian Ocean more than 16 MYA, diversified there and gave rise to the Aegypti Group, before entering continental Africa as the *Ae. aegypti* s.s. lineage.

Our analyses on the divergence times of species in the Aegypti Group on Indian Ocean islands (Figure 2) correspond well with the ages of the islands on which these species are found, as the age of Mayotte (where *Ae. pia* is endemic) is less than 20 MY (Michon, 2016) and Mauritius (where *Ae. mascarensis* is endemic) is less than 9 MY (Ashwal, Wiedenbeck, & Torsvik, 2017; Warren, Bermingham, Bowie, Prys‐Jones, & Thébaud, 2003). Our analyses support that ancestral populations in the Aegypti Group on Madagascar diverged prior to the divergence of *Ae. mascarensis* on Mauritius, east of Madagascar, a result consistent with previous single‐marker analysis with limited taxon sampling that found populations in the Aegypti Group on Madagascar were basal to continental African *Aedes aegypti* (Delatte et al., 2011). This observation is also consistent with the maximum‐likelihood analysis on the primary SNP data set reported here, although branch lengths from our SNP‐based likelihood analysis may be underestimating variation in more distant species and populations, as the SNP chip was designed to capture variation within *Ae. aegypti*. Nonetheless, the concordance between the analyses performed on our nucleotide sequence data set, our primary SNP dataset and the microsatellite estimation of population history (see below) indicates that the common ancestor of the Aegypti Group was most likely found throughout the islands in the southwestern Indian Ocean.

Our results suggest that *Ae. aegypti* s.s. entered Africa less than 85,000 years ago, as the divergence of Europa and continental African populations was younger than 85,000 years across all five ABC analyses (Figure S3; Tables S14 and S15). Pollen analyses of cores from Lake Malawi in East Africa at about the same latitude as Madagascar suggest frequent fluctuations in dry‐ and wet‐associated flora until about 80,000 years ago when present‐day vegetation stabilized (Ivory, Lézine, Vincens, & Cohen, 2018; Lyons et al., 2015). This is consistent with the unusually high and stable levels that Lake Malawi experienced over the last 70,000 years, indicative of a period of steady rain (Ivory et al., 2018; Lyons et al., 2015) that would have provided a suitable habitat typical of Aaf on continental Africa.

Interestingly, our F3 tests did not indicate admixture has occurred in *Ae. aegypti* from Europa, suggesting that the apparent admixture signatures detected by the STRUCTURE‐like SNMF in this population (Figure S4) may represent instead ancestral polymorphism for this lineage. If true, *Ae. aegypti* from Europa could be a remnant of the island‐dwelling lineage that originally colonized continental Africa, although testing this hypothesis requires further sampling and analysis. Moreover, we estimated a strikingly early last common ancestor for continental *Ae. aegypti* s.s. in Africa, at approximately 17,000 to 25,000 years ago. This time period coincides with the end of the last glacial maximum (Clark et al., 2009), where stable Pleistocene forest refugia along the east African coast (Finch, Leng, & Marchant, 2009; Fjeldsaå & Lovett, 1997; Mumbi, Marchant, Hooghiemstra, & Wooller, 2008) may have provided suitable habitat for *Ae. aegypti* to more widely disperse across Africa. Following the end of the last glacial maximum, forests spread across Africa and reached their extent approximately 6,000 years ago, covering most of central Africa (Hoelzmann et al., 1998; Watrin, Lézine, & Hély, 2009) and enabling dispersal westward. Our estimates for the divergence of East and West African populations of *Ae. aegypti* are consistent with Bennett et al. (2016) who presented molecular data suggesting the deepest divergences of populations of *Aedes aegypti* on continental Africa occurred between 6,000 and 120,000 years ago. Much more recently, the continental *Ae. aegypti* s.s. lineage separated into the two recognized subspecies *Ae. aegypti aegypti* (Aaa) and *Ae. aegypti formosus* (Aaf). This has been speculated to have begun when human settlements developed in sub‐Saharan Africa and water was stored during the dry season. The full separation of subspecies occurred ~500 years when European slave trade brought *Ae. aegypti* s.s. to the New World beginning in the 16th century (Powell et al., 2018).

### 4.2 A divergent lineage of *Aedes aegypti* on Madagascar

We find strong evidence that *Ae. aegypti* on Madagascar are genetically distinct, and deeply diverged from *Ae. aegypti* s.s., at a level equal to or greater than what is seen in the named species *Ae. mascarensis* (Figures 2 and 3). A previous study, including only putative *Ae. aegypti* samples, suggested Madagascar *Ae. aegypti* separated from Europa and continental populations 7–15 MYA (Fort et al., 2012), similar to our estimate (Figure 2). Phylogenetic analyses using mtDNA from mosquitoes from northern Madagascar that were morphologically identified as Aaf indicated that these mosquitoes were genetically distinct from other populations of *Ae. aegypti* (Mousson et al., 2005), and basal to *Ae. mascarensis*, consistent with our results. To date, no phenotypic differences are known between *Ae. aegypti* from Madagascar and *Ae. aegypti* s.s., but ecological observations of *Ae. aegypti* on Madagascar suggest a sylvan mosquito similar in appearance and behaviour to Aaf (Fontenille & Rodhain, 1989; Raharimalala et al., 2012), with differences in vector competence relative to *Ae. aegypti* s.s. (Failloux et al., 2002; Vazeille et al., 2001). A cryptic lineage—particularly one that has shown some evidence of differences in vector competence relative to *Ae. aegypti* s.s.—may present a unique challenge to control efforts. Surveillance efforts may need to account for divergent lineage, whose risk to public health may be significantly different from *Ae. aegypti* s.s., made doubly hard by the cryptic nature of this taxa and its largely unknown ecology. However, in the absence of tests for reproductive isolation and detailed morphological analysis, it seems premature at this time to formally describe a new species. Furthermore, it is important to note that the sample from Madagascar we analysed came from a single locality. Madagascar is well known as a biodiversity hot spot, and thus, it is conceivable, maybe even likely, that other cryptic taxa in the Aegypti Group occur on Madagascar. This is especially true as two sources of molecular data (Fort et al. and nucleotide data from this study) indicate these populations have been on Madagascar for >7 million years.

### 4.3 A recent history of introgression in *Ae. aegypti*

On the islands in the southwestern Indian Ocean, we find evidence for recent introgression among members of the Aegypti Group. These results indicate a component of the admixture is Aaa from Asia, rather than from New World Aaa or from Aaf. In our analysis, Aaa from Saudi Arabia was most often identified as the Aaa parental population in admixture analyses, but we note that the inference of ancestral population origins with Treemix is limited to the populations used in the analysis, and thus, future sampling may yield different results; additionally, all Asian populations we tested had similar strength of support as Saudi Arabia to be the origin of the parental Aaa involved in introgression in these island populations (Tables S13–S15). This inference is consistent with a hypothesis of Aaa expanding eastward after the Suez Canal opened in 1869 to colonize Asia (Kotsakiozi, Gloria‐Soria, Schaffner, Robert, & Powell, 2018; Powell et al., 2018). Here, we infer they also entered islands east of Africa where they encountered other species in the Aegypti Group and, in some cases, interbred. It is also possible due to the small sample sizes in some populations (e.g. *Ae. mascarensis* or *Ae. aegypti* from Sri Lanka) we have underestimated or missed potential admixture events, as the relative F3 statistics inferred from our analysis of resampled populations were slightly lower than those from the full SNP data set. However, our resampling provided qualitatively the same results as our full data set. This indicates that small sample sizes can detect admixture that is consistent with observations from larger samples of the same population, at least given the relatively large number of SNPs in our study.

The consequences of this introgression remain to be tested. However, some *Ae. aegypti* on Réunion exhibit a particular scutal morph that appears intermediate between *Ae. aegypti* and *Ae. mascarensis* (Le Goff et al., in preparation). Other *Ae. aegypti* from Réunion—differentiated from Aaf in scaling patterns—have been reported to be primarily a sylvatic mosquito (Bagny, Delatte, Elissa, Quilici, & Fontenille, 2009; Salvan & Mouchet, 1994); that is, most are morphologically Aaa but ecologically Aaf. Previous studies speculated that the presence of Aaa in rural locations on Réunion, rather than in urban locations typical of Aaa, was due to the widespread use of pesticides, and larval competition with *Ae. albopictus* (Bagny et al., 2009). We propose here that admixture with *Ae. mascarensis*, or a mascarensis‐like population, could have resulted in behavioural shifts due to the sylvan nature of *Ae. mascarensis*, while the morphology typical of Aaa was maintained. Admixture may complicate efforts to control *Ae. aegypti* by shifting typical behaviours associated with this mosquito in a way that may allow them to evade control measures that are typically effective against them. Vector control efforts in this region thus may need to account for not only a cryptic species, but also admixed populations, and will likely require more detailed information on ecological differences between species and populations to be successful. Moreover, the primary vector of the chikungunya virus during the outbreak of 2005/6 on Réunion was attributed to *Ae. albopictus* (Delatte et al., 2008), rather than *Ae. aegypti*, the more competent vector present throughout the rest of the world. Does this mean *Ae. aegypti* on Réunion is less competent than most *Ae. aegypti* populations for chikungunya? While some information is available for Madagascar (Failloux et al., 2002; Vazeille et al., 2001), the vector competence of other members of the Aegypti Group on southwestern Indian Ocean islands is largely unknown.

## 5 CONCLUSION

*Ae. aegypti* is among the best‐studied mosquitoes, yet from an evolutionary standpoint, the results presented here highlight how much more there is still to be learned. We present the first solid evidence that the common ancestor *Ae. aegypti* s.s. and its nearest relatives were occupying Madagascar and/or islands in the southwestern Indian Ocean, indicating that these islands gave rise only relatively recently (<85,000 YA) to *Ae. aegypti* s.s. on continental Africa. Moreover, we find strong evidence of a cryptic genetic lineage on Madagascar. It is also clear, however, that while our limited sampling in this region of the world has revealed important insights, more intense sampling and analyses of collections are likely to reveal a more complete picture of the evolutionary history of the Aegypti Group.

## AUTHOR CONTRIBUTIONS

JS, AGS and JRP designed research. JS, AGS, VR and GLG performed research. AGS, VR, GLG, ABF and JRP contributed samples. JS and AGS analysed data. JS, AGS, VR, GLG, ABF and JRP wrote the manuscript.

## Supporting information

Supplementary Material

## Data Availability

The nucleotide alignment in Nexus format is available at FigShare.com, https://doi.org/10.6084/m9.figshare.12476189. VCF file containing SNP calls is available at VectorBase.org, Population Biology Project ID: VBP0000672. Microsatellite raw allele frequencies are available at VectorBase.org, Population Biology Project ID: VBP0000672. [dataset] Soghigian, Gloria‐Soria, Robert, Le Goff, Failloux, and Powell; 2020; VCF SNP calls for Ae. aegypti and related species from the southwestern Indian Ocean; Vector Base; Population Biology Project identifier VBP0000672. [dataset] Soghigian, Gloria‐Soria, Robert, Le Goff, Failloux, and Powell; 2020; Raw allele frequencies for Ae. aegypti from the southwestern Indian Ocean; Vector Base; Population Biology Project VBP0000672. [dataset] Soghigian, Gloria‐Soria, Robert, Le Goff, Failloux, and Powell; 2020; Nucleotide alignment for *Ae. aegypti* and other Culicidae; Fig Share; https://doi.org/10.6084/m9.figshare.12476189.
