# Supplementary material for: Genetic evidence for the origin of Aedes aegypti, the yellow fever mosquito, in the southwestern Indian Ocean
Source: Mol Ecol. 2020 Aug 30;29(19):3593–606. doi: 10.1111/mec.15590 (PMC7589284; doi:10.1111/mec.15590)
Supplement: Supplementary file 1 — Supplementary Material [file MEC-29-3593-s001.pdf]

Supplemental Text, Figures, and Tables for "Genetic evidence for the origin of *Aedes aegypti*, the yellow fever mosquito, in the southwestern Indian Ocean"

John Soghigian [1,2*], Andrea Gloria-Soria [1,3], Vincent Robert [4], Gilbert Le Goff [4], Anna-Bella Failloux [5], Jeffrey R. Powell[1]

[1] Yale University, 21 Sachem Street, New Haven, CT 06520-8105 USA

[2] Department of Entomology and Plant Pathology, North Carolina State University, Raleigh, NC, 27603

[3] Center for Vector Biology & Zoonotic Diseases. Department of Environmental Sciences. The Connecticut Agricultural Experiment Station. 123 Huntington St., New Haven, CT 06504

[4] MIVEGEC Unit, IRD, Montpellier Univ., CNRS, Montpellier, France

[5] Institut Pasteur, Arboviruses and Insect Vectors, 25 rue du Dr Roux, F-75724, Paris, France

*Corresponding Author : john.soghigian@gmail.com

Contents

Supplemental Text 2
Supplemental Figures 3
- Figure S1 4
- Figure S2 6
- Figure S3 7
- Figure S4 8
- Figure S5 9

Supplemental Tables 10
- Table S1 10
- Table S2 11
- Table S3 12
- Table S4 13
- Table S5 14
- Table S6 15
- Table S7 16
- Table S8 17
- Table S9 18
- Table S10 19
- Table S11 20
- Table S12 21
- Table S13 22
- Table S14 23
- Table S15 25
- Table S16 28
- Table S17 29

Supplemental Text

Missing Data

As we were overrode default settings for missing data in order to call SNPs for *Aedes pia*, we also investigated the association between missing data and population differentiation.

Analysis of Missing Data

We calculated the proportion of missing data per individual from each population (or species for *Aedes pia* and *Aedes mascarensis*). We tested whether there was a significant difference among populations in amount of missing data with an ANOVA, and then used a Tukey's HSD to assess where significant differences occurred in R.

Results of Missing Data Anslysis

The amount of missing data present per population showed additional signs of the distinctiveness – and potential admixture – of island populations, when genotyped on the SNP chip designed for *Ae. aegypti* s.s. (Figure S5). We expect the efficiency of the probes designed for *Ae. aegypti* s.s. to decline with the genetic distance of the species being tested given the probes were designed from genomic sequencing of *Aedes aegypti aegypti*.

There was a significant difference in missing data among populations ($F_{18,180}$=122.2, P<0.001; Table S17), but this missing data difference was only significant among divergent sibling species and island populations (Fig S5). Moreover, the proportion of missing data is consistent with approximate genetic distance in our phylogenetic analyses (Figure 4); populations more distant from Aaa/Aaf have proportionally more missing data, with the greatest missing data observed in *Ae. pia*, followed by *Ae. mascarensis* and *Aedes aegypti* from Madagascar. Furthermore, the island populations of Reunion and Mayotte appear to be intermediates between *Ae. mascarensis, Ae. aegypti* from Madagascar, and *Ae. aegypti* s.s.; this higher proportion of missing data could thus be a result of admixture, an observation consistent with our SNMF (Figure S4) and our tests of admixture in Treemix.

Supplemental Figures

Figure S1

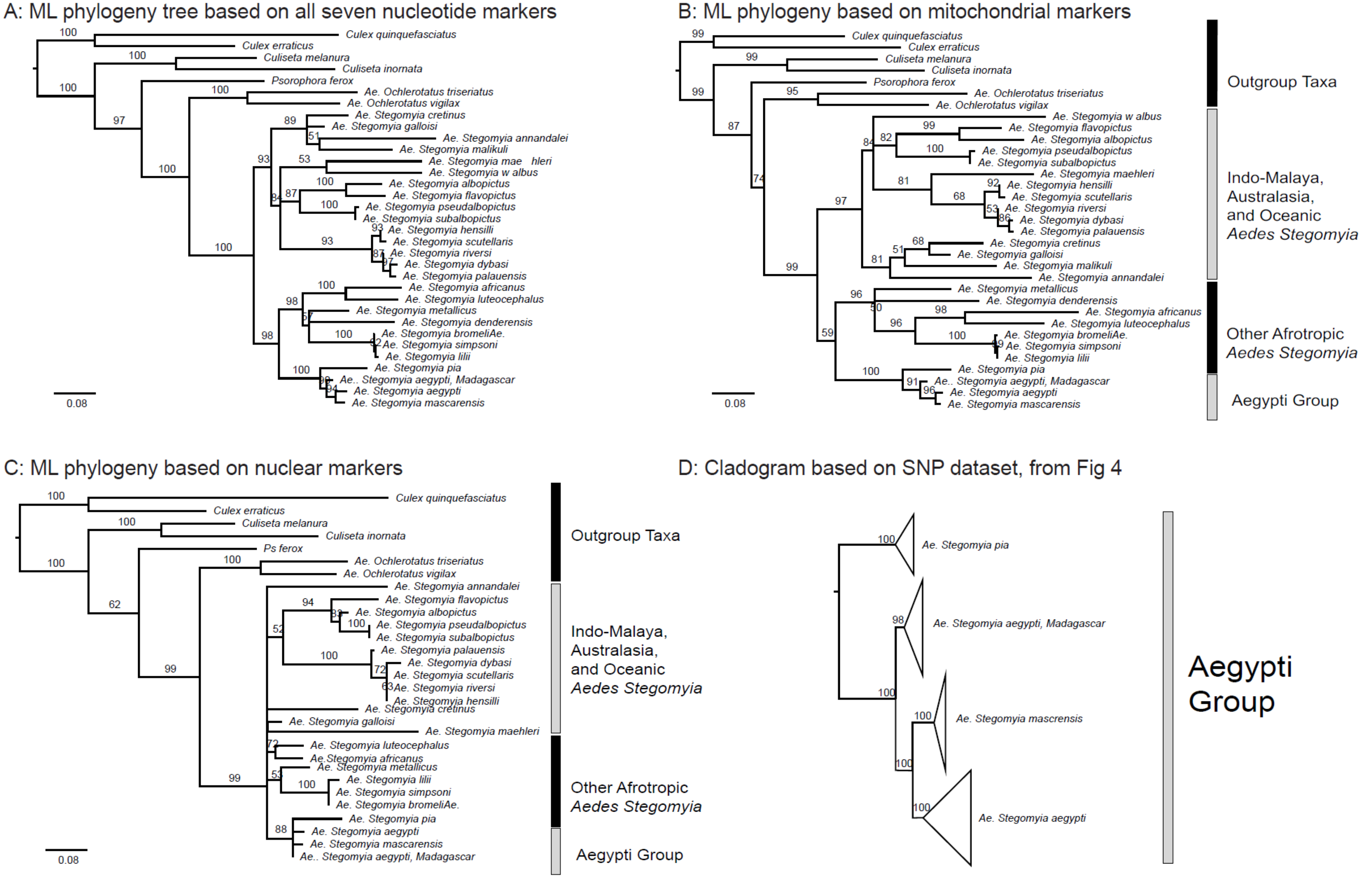
A: ML phylogeny tree based on all seven nucleotide markers
B: ML phylogeny based on mitochondrial markers
C: ML phylogeny based on nuclear markers
D: Cladogram based on SNP dataset, from Fig 4
Outgroup Taxa
Indo-Malaya, Australasia, and Oceanic *Aedes Stegomyia*
Other Afrotropic *Aedes Stegomyia*
Aegypti Group
Culex quinquefasciatus
Culex erraticus
Culiseta melanura
Culiseta inornata
Psorophora ferox
Ae. Ochlerotatus triseriatus
Ae. Ochlerotatus vigilax
Ae. Stegomyia pia
Ae. Stegomyia aegypti, Madagascar
Ae. Stegomyia mascrensis
Ae. Stegomyia aegypti
0.08

Fig S1. Maximum likelihood phylogenetic analyses in IQ-Tree recover concordant topologies for clades with high support values across A) the full seven marker nucleotide dataset, B) the nucleotide dataset subset to mitochondrial markers alone, C) the nucleotide dataset subset to nuclear markers alone, and D) the 18,849 SNP dataset, represented here as a cladogram of relationships between species in the Aegypti Group, from Figure 4 in the main manuscript text. Branch lengths are not to scale in Panel D (see Figure 4 in main manuscript text). Branch support values are UFBoot values from IQ-Tree. Branches collapsed at extremely poorly supported nodes for comparison (<50).

Figure S2

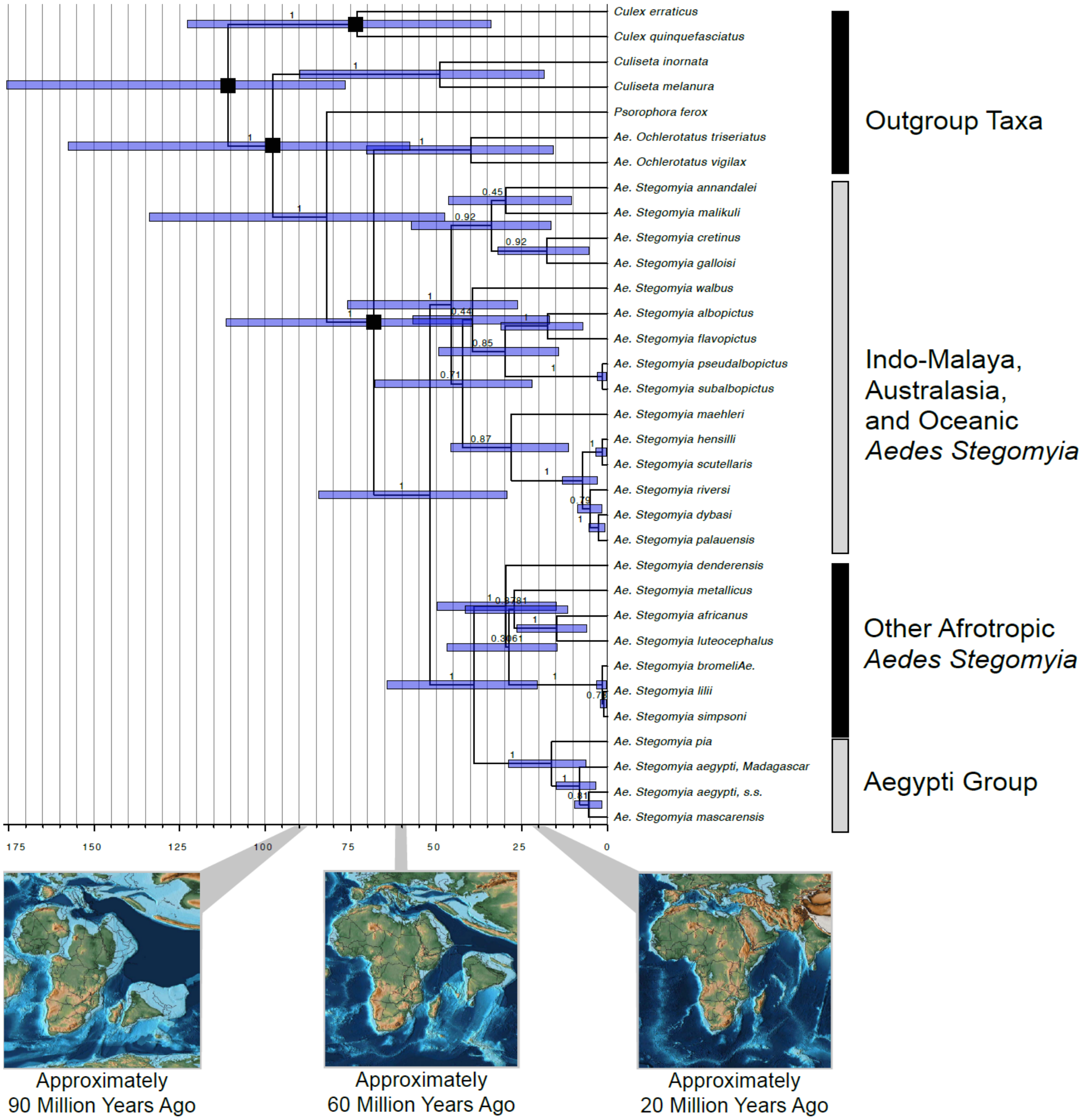

Fig. S2. Full analysis of divergence times in the subgenus *Stegomyia* from the seven marker nucleotide dataset in BEAST2. Values on branches are posterior probabilities. Bars indicate 95% HPDs. Black boxes indicate the location of fossil-based constraints; see Supplementary Table 6 for more information.

Figure S3

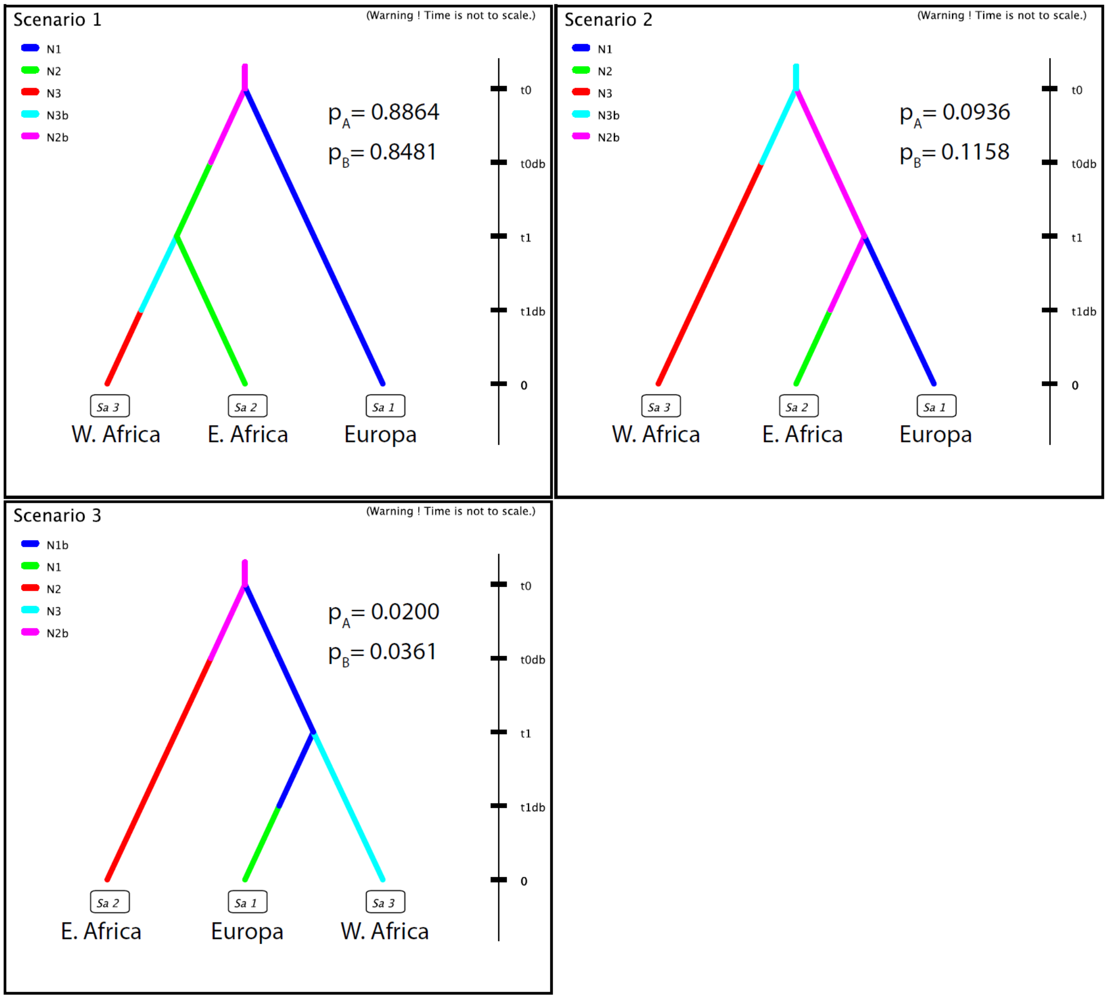

Fig. S3. Visual depiction of the approximate Bayesian computation (ABC) analyses across two runs (a and b) with separate populations from East and West Africa in each of the runs. Across runs, the best-supported scenario was Scenario 1, where Europa diverged prior to Africa. See Supplemental Table 1 and 2 for additional details on these results.

Figure S4

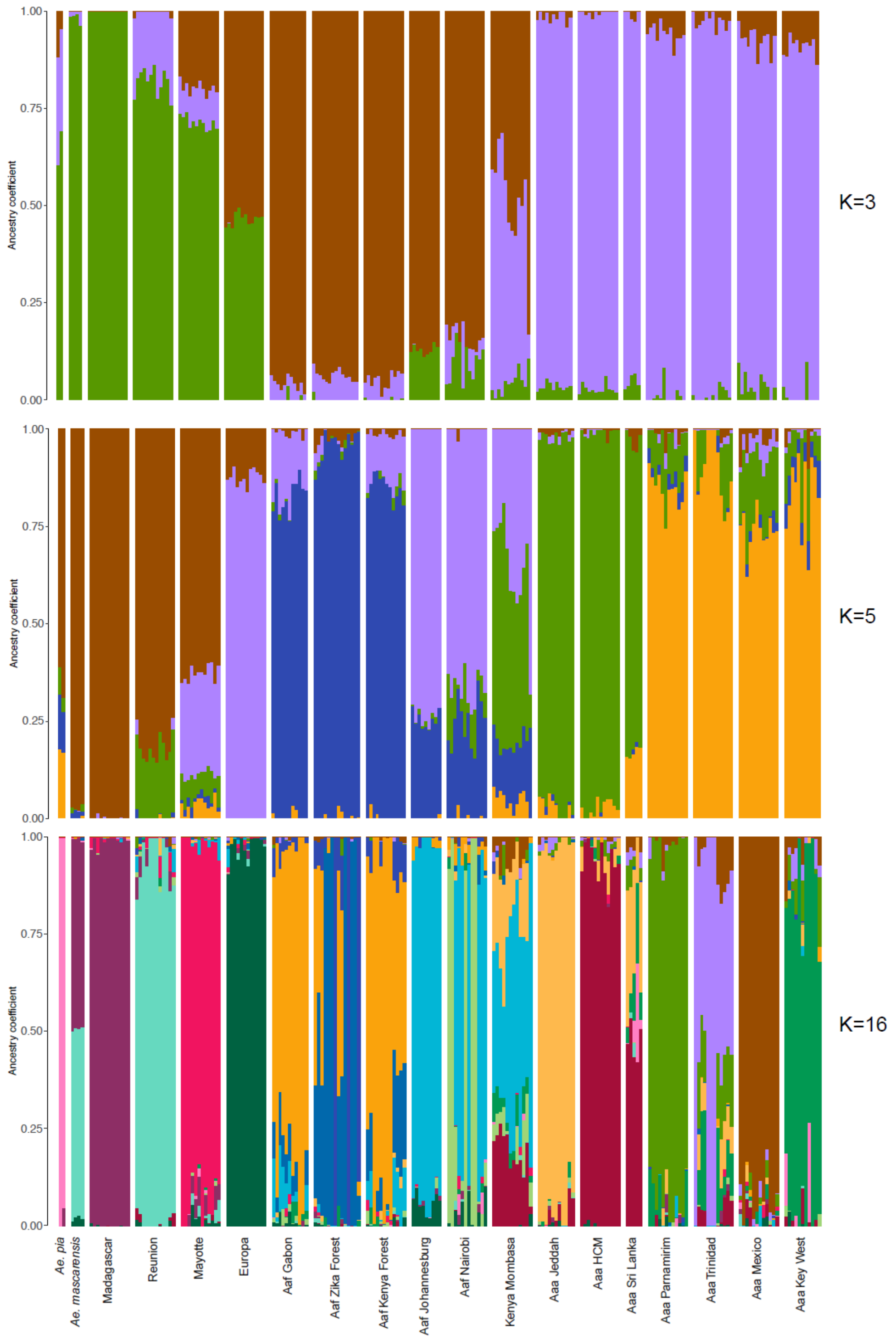

Fig. S4. STRUCTURE-like SNMF analysis of the Aegypti Group SNP dataset demonstrates differentiation among subspecies of *Aedes aegypti* and the islands in the southwestern Indian Ocean.

Figure S5

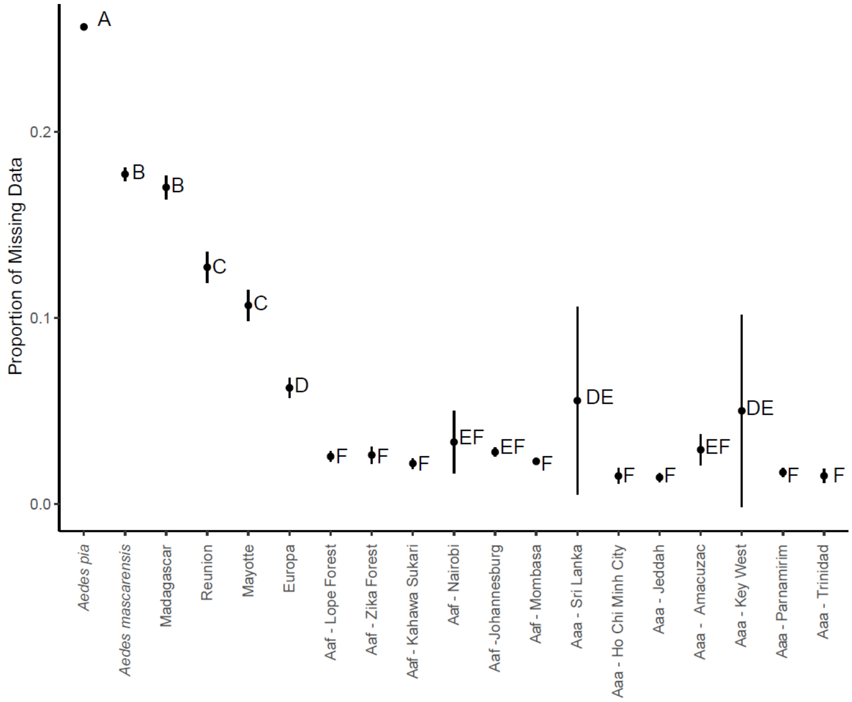

Fig. S5. *Aedes aegypti* from Madagascar appear as distant as *Aedes mascarensis* from *Aedes aegypti* s.s., with regards to missing data. Putatively admixed populations show intermediate levels of missing data. Mean missing data in the Aegypti Group SNP dataset for each population and subspecies. Error bars are standard errors and different letters are significantly different from one another according to a posthoc Tukey's HSD (see Supplemental Tables).

Supplemental Tables

Table S1: Geographic sampling location of species and populations of *Aedes aegypti* used in this study.

Subspecies/Region	Geographic Sampling Location	N	Year Sampled*	Originating Study
Ae. mascarensis	Mauritius	4		see Kotsakiozi et al. 2018
Ae. pia	Mayotte	2	2011	This study
Ae. aegypti aegypti	Ho Chi Min, Vietnam	12		see Gloria-Soria et al. 2018
Ae. aegypti aegypti	Jeddah, Saudi Arabia	11		see Gloria-Soria et al. 2018
Ae. aegypti aegypti	Amacuzac, Mexico	12		see Gloria-Soria et al. 2018
Ae. aegypti aegypti	Key West, Florida	11		see Gloria-Soria et al. 2018
Ae. aegypti aegypti	Parnamirim, Brazil	12	2012	This study
Ae. aegypti aegypti	Sri Lanka	5		see Gloria-Soria et al. 2018
Ae. aegypti aegypti	Trinidad	12		see Gloria-Soria et al. 2018
Ae. aegypti formosus	Lope Forest, Gabon	12		see Kotsakiozi et al. 2018
Ae. aegypti formosus	Kahawa Sukari, Kenya	12		see Kotsakiozi et al. 2018
Ae. aegypti formosus	Mombasa, Kenya	12	2017	This study
Ae. aegypti formosus	Nairobi, Kenya	12		see Kotsakiozi et al. 2018
Ae. aegypti formosus	Zika Forest, Uganda	14		see Kotsakiozi et al. 2018
Ae. aegypti formosus	Johannesburg, South Africa	9		see Gloria-Soria et al. 2018
Ae. aegypti - Island	Europa	12	2008	This study
Ae. aegypti - Island	Mahaleja, Madagascar	12	2011	This study
Ae. aegypti - Island	Mayotte	12	2011	This study
Ae. aegypti - Island	Reunion	12		see Kotsakiozi et al. 2018

*Listed for this study. See publishing study for additional informational on previously published samples.

Table S2: Presence, indicated by X, and absence of markers, per taxon, in our nucleotide sequence alignments. Species groups within the subgenus *Stegomyia* are indicated. Members of the Aegypti Group are bolded. Taxa with * are newly sequenced for this study.

Species	*Stegomyia* Group[a]	18S	28S	Arginine Kinase	COI	COII	Enolase	ITS2
*Ae. Ochlerotatus triseriatus**		X	X	X	X	X	X	X
*Ae. Ochlerotatus vigilax**		X	X	X	X	X	X	
Ae. Stegomyia aegypti	**Aegypti**	**X**	**X**	**X**	**X**	**X**	**X**	**X**
Ae. Stegomyoa 'aegypti' Madagascar*	**Aegypti**	**X**	**X**	**X**	**X**	**X**		**X**
Ae. Stegomyia albopictus	Albopictus[b]	X	X	X	X	X	X	X
Ae. Stegomyia annandalei	Annandalei[c]		X		X	X		
Ae. Stegomyia bromeliae	Simpsoni	X	X	X	X		X	X
Ae. Stegomyia cretinus	Unilineatus				X			X
Ae. Stegomyia denderensis	Apicoargenteus				X			
Ae. Stegomyia dybasi	Scutellaris[b]		X		X			
Ae. Stegomyia flavopictus	Albopictus[b]		X		X			X
Ae. Stegomyia galloisi	Albopictus[b]		X		X			
Ae. Stegomyia hensilli	Scutellaris[b]		X		X			
Ae. Stegomyia lilii	Simpsoni				X			X
Ae. Stegomyia luteocephalus	Africanus	X	X	X	X	X	X	X
Ae. Stegomyia maehleri	Maehleri		X		X			
Ae. Stegomyia malikuli	Meiopunctatus[c]				X	X		
Ae. Stegomyia mascarensis	**Aegypti**	**X**	**X**	**X**	**X**	**X**	**X**	**X**
Ae. Stegomyia metallicus	Metallicus	X	X	X	X		X	X
Ae. Stegomyia palauensis	Scutellaris[b]		X		X			
Ae. Stegomyia pia	**Aegypti**	**X**	**X**		**X**			**X**
Ae. Stegomyia pseudalbopictus	Albopictus[b]		X		X			
Ae. Stegomyia riversi	Scutellaris[b]		X		X			X
Ae. Stegomyia scutellaris	Scutellaris[b]		X	X	X			
Ae. Stegomyia simpsoni	Simpsoni			X	X			X
Ae. Stegomyia subalbopictus	Albopictus[b]		X	X	X	X		
Ae. Stegomyia w-albus	W-albus[c]				X			
Ae. Stegomyia africanus	Africanus	X	X	X	X		X	
*Culex erraticus**		X	X	X	X	X	X	X
Culex quinquefasciatus		X	X	X	X	X	X	X
*Culiseta inornate**		X	X	X	X	X	X	X
*Culiseta melanura**		X	X	X	X	X		X
Psorophora ferox		X	X	X	X	X	X	X

a: The Species Group to which each *Stegomyia* species belongs, following the Mosquito Taxonomic Inventory (http://mosquito-taxonomic-inventory.info/, retrieved 6/1/2020).

b: Albopictus and Scutellaris are subgroups of the Scutellaris group.

c: Annandelei, Meiopunctatus, and W-albus are subgroups of the W-albus group.

Table S3: Marker name and primer sequences (5' to 3') used to amplify nucleotide sequence data reported here. The original source reference for each primer is provided here, and see Soghigian et al. 2017 for additional details.

Marker	Primer sequence	Reference
18S - Small Ribosomal Subunit	F- GTTGAGTCAAATTAAGCCGC<br>R - CTGCCCTATCAACTATTGATGG	Soghigian et al. 2017
28S - Large Ribosomal Subunit	F- GGAGTCGTGTTGCTTGATA<br>R - CCAGCTACTAGATGGTTCG	Soghigian et al. 2017
Arginine Kinase	F – GCTTCAAGAAGACCGACAAGCAC<br>R – GCCATCGTACATCTCCTTGACG	Reidenbach et al. 2009
COI - Cytochrome Oxidase Subunit I	F - GGTCAACAAATCATAAAGATATTGG<br>R – TAAACTTCAGGGTGACCAAAAAATCA	Folmer et al. 1994
COII - Cytochrome Oxidase Subunit II	F - ATGGCAGATTAGTGCAATGA<br>R - GTTTAAGAGACCAGTACTTG	Cook et al. 2005
Enolase	F - AGRATYTGGTTGTACTTGGC<br>R - ATGCAGGAGTTCATGATCCTG	Reidenbach et al. 2009
ITS2 - Internal Transcribed Spacer II	F – TGTGAACTGCAGGACACATG<br>R - TCACACATTATTTGAGGCCTAC	Wesson et al. 1992

`

Table S4: A nested analysis of molecular variance (AMOVA) using species/subspecies and population of origin.

	Df	Sum of Squares	Mean Square	Perc. Var.*	P-value**
Between subspecies/species	3	123669.19	41223.06	32.65	0.001
Among samples within a subspecies/species	15	93957.36	6263.82	19.45	0.001
Within samples	180	210852.05	1171.40	47.90	0.001
Total	198	428478.60	2164.03		

*Percent of variance (out of 100) refers to the proportion of variance attributed to that component in the AMOVA

**P-value calculated from a randomization test with 999 permutations.

Table S5: Pairwise Fst values among regions included in this study

	Aaa	Aaf	Islands	*Ae. pia*
Ae. mascarensis	0.38	0.28	0.14	0.53
Ae. pia	0.39	0.36	0.39	
Islands	0.32	0.17		
Aaf	0.22			

All pairwise Fst values are significant ($P<0.05$). For populations included in Islands, Aaf, and Aaa categories, see Table 1.

Table S6: Pairwise Fst values among populations

	1	2	3	4	5	6	7	8	9	10	11	12	13	14	15	16	17	18
1. *Ae. mascarensis*																		
2. *Ae. pia*	0.53																	
3. Europa	0.56	0.63																
4. Lope Forest, Gabon	0.39	0.43	0.36															
5. Ho Chi Min, Vietnam	0.48	0.45	0.52	0.35														
6. Jeddah, Saudi Arabia	0.50	0.47	0.54	0.36	0.12													
7. Kahawa Sukari, Kenya	0.37	0.42	0.34	0.06	0.34	0.35												
8. Mombasa, Kenya	0.33	0.34	0.33	0.16	0.17	0.17	0.15											
9. Nairobi, Kenya	0.36	0.41	0.29	0.14	0.34	0.35	0.13	0.11										
10. Madagascar	0.34	0.57	0.54	0.44	0.55	0.57	0.42	0.40	0.41									
11. Mayotte	0.21	0.38	0.33	0.25	0.37	0.39	0.24	0.21	0.21	0.22								
12. Amacuzac, Mexico	0.46	0.43	0.51	0.33	0.16	0.17	0.31	0.17	0.32	0.53	0.36							
13. Key West, Florida	0.45	0.40	0.49	0.30	0.14	0.16	0.29	0.15	0.30	0.52	0.34	0.11						
14 Parmir, Brazil	0.49	0.46	0.53	0.34	0.16	0.17	0.33	0.18	0.34	0.55	0.38	0.14	0.11					
15. Reunion Island	0.22	0.45	0.49	0.37	0.40	0.41	0.35	0.28	0.34	0.36	0.22	0.39	0.38	0.41				
16. Johannesburg, South Africa	0.43	0.47	0.32	0.16	0.39	0.41	0.16	0.15	0.05	0.47	0.24	0.37	0.35	0.39	0.39			
17. Sri Lanka	0.52	0.42	0.58	0.35	0.09	0.11	0.33	0.15	0.33	0.59	0.37	0.15	0.13	0.15	0.41	0.40		
18. Trinidad	0.50	0.47	0.54	0.36	0.17	0.18	0.34	0.20	0.35	0.56	0.39	0.14	0.12	0.13	0.43	0.41	0.16	
19. Zika Forest, Uganda	0.39	0.43	0.37	0.06	0.35	0.36	0.05	0.18	0.17	0.43	0.27	0.33	0.30	0.34	0.37	0.20	0.35	0.35

Table S7: The top 5 Admixture Scenarios For Mombasa, Mayotte, and Reunion, sorted by z score, from the full dataset. Top hit for each admixed population is bolded for comparison, but all listed scenarios have a high probability

Pop	Anc1	Anc2	F3	std_error	z	P-value
**Kenya_Mombasa**	**SAfrica_Johannesburg**	**Jeddah**	-0.020	**4.89E-04**	**-41.77**	**0**
Kenya_Mombasa	Ho_Chi_Minh_City	SAfrica_Johannesburg	-0.020	4.81E-04	-41.59	0
Kenya_Mombasa	Sri_Lanka	SAfrica_Johannesburg	-0.020	4.91E-04	-40.63	0
Kenya_Mombasa	Kenya_Nairobi	Jeddah	-0.016	4.23E-04	-37.99	0
Kenya_Mombasa	Ho_Chi_Minh_City	Kenya_Nairobi	-0.016	4.16E-04	-37.63	0
**Mayotte**	**Madagascar**	**Jeddah**	-0.013	**6.04E-04**	**-21.35**	**5.58E-101**
Mayotte	Madagascar	Ho_Chi_Minh_City	-0.013	6.06E-04	-21.25	3.88E-100
Mayotte	Madagascar	Kenya_Mombasa	-0.010	4.65E-04	-20.53	1.64E-93
Mayotte	Madagascar	Sri_Lanka	-0.012	6.02E-04	-19.88	7.71E-88
Mayotte	North_KeyWest	Madagascar	-0.011	5.80E-04	-19.71	2.30E-86
**Reunion**	***Aedes_mascarensis***	**Jeddah**	-0.007	**7.31E-04**	**-10.09**	**6.36E-24**
Reunion	*Aedes_mascarensis*	Ho_Chi_Minh_City	-0.007	7.33E-04	-9.43	4.37E-21
Reunion	Sri_Lanka	*Aedes_mascarensis*	-0.006	7.43E-04	-8.32	9.42E-17
Reunion	*Aedes_mascarensis*	Parnamirim	-0.005	7.43E-04	-7.19	6.89E-13
Reunion	*Aedes_mascarensis*	Mexico_Amacuzac	-0.005	7.19E-04	-7.07	1.61E-12

Table S8: The top 5 Admixture Scenarios For Mombasa, Mayotte, and Reunion, sorted by z score, from the SNP dataset filtered for completeness. Top hit for each admixed population is bolded for comparison, but all listed scenarios have a high probability.

Pop	Anc1	Anc2	F3	std_error	z	P-value
**Kenya_Mombasa**	**Kenya_Nairobi**	**Jeddah**	**-0.015**	**0.001**	**-21.250**	**3.19E-99**
Kenya_Mombasa	Sri_Lanka	Kenya_Nairobi	-0.015	0.001	-18.581	3.18E-76
Kenya_Mombasa	Sri_Lanka	SAfrica_Johannesburg	-0.019	0.001	-17.678	3.78E-69
Kenya_Mombasa	Ho_Chi_Minh_City	SAfrica_Johannesburg	-0.018	0.001	-15.480	2.04E-53
Kenya_Mombasa	Ho_Chi_Minh_City	Kenya_Nairobi	-0.014	0.001	-15.061	1.23E-50
**Mayotte**	**Madagascar**	**Jeddah**	**-0.011**	**0.002**	**-6.867**	**9.68E-12**
Mayotte	Madagascar	Mexico_Amacuzac	-0.009	0.001	-6.284	4.63E-10
Mayotte	Madagascar	Ho_Chi_Minh_City	-0.010	0.002	-6.101	1.46E-09
Mayotte	Madagascar	Sri_Lanka	-0.010	0.002	-6.093	1.53E-09
Mayotte	Madagascar	Trinidad	-0.009	0.002	-5.920	4.38E-09
**Reunion**	**Aedes_mascarensis**	**Jeddah**	**-0.010**	**0.002**	**-4.921**	**1.05E-06**
Reunion	Sri_Lanka	Aedes_mascarensis	-0.009	0.002	-4.763	2.30E-06
Reunion	Aedes_mascarensis	Ho_Chi_Minh_City	-0.009	0.002	-4.562	5.96E-06
Reunion	Aedes_mascarensis	Parnamirim	-0.008	0.002	-4.343	1.62E-05

Table S9: The top 5 Admixture Scenarios For Mombasa, Mayotte, and Reunion, where we resampled *Ae. aegypti* populations down to 4 individuals each 100 times. Top hit for each admixed population is bolded for comparison, but all listed scenarios have a high probability. This table is sorted by F3 score.

Pop	Anc1	Anc2	F3	Lower 95% Bound	Upper 95% Bound
**Kenya_Mombasa**	**Jeddah**	**SAfrica_Johannesburg**	**-0.008**	**-0.013**	**-0.003**
Kenya_Mombasa	Ho_Chi_Minh_City	SAfrica_Johannesburg	-0.008	-0.013	-0.003
Kenya_Mombasa	Jeddah	Kenya_Nairobi	-0.006	-0.011	-0.001
Kenya_Mombasa	Kenya_Nairobi	Sri_Lanka	-0.005	-0.009	-0.001
Kenya_Mombasa	Ho_Chi_Minh_City	Kenya_Nairobi	-0.005	-0.010	-0.001
**Mayotte**	**Jeddah**	**Madagascar**	**-0.003**	**-0.006**	**0.000**
Mayotte	Ho_Chi_Minh_City	Madagascar	-0.002	-0.005	0.001
Mayotte	Sri_Lanka	Madagascar	-0.002	-0.005	0.002
Mayotte	Mexico_Amacuzac	Madagascar	-0.001	-0.004	0.002
Mayotte	Parnamirim	Madagascar	-0.001	-0.004	0.002
**Reunion**	**Jeddah**	**Aedes_mascarensis**	**-0.004**	**-0.009**	**0.000**
Reunion	Ho_Chi_Minh_City	Aedes_mascarensis	-0.003	-0.008	0.000
Reunion	Sri_Lanka	Aedes_mascarensis	-0.003	-0.008	0.002
Reunion	Trinidad	Aedes_mascarensis	-0.002	-0.007	0.002
Reunion	Parnamirim	Aedes_mascarensis	-0.002	-0.007	0.003

Table S10: Alleles per microsatellite locus

Locus	Alleles
AC1	19
AC2	17
AC4	9
AC5	21
CT2	13
AG1	14
AG2	10
AG5	13
A1	10
A9	13
B2	9
B3	11
TOTAL	159

Table S11: Microsatellite dataset characteristics, per population.

Population	% Polymorphic Loci	% Missing data per locus
Europa	91.67%	0
Ngari	75.00%	%25 (AC4); 100% (A9)
Cameroon	100.00%	0
Gabon	100.00%	10% (A9)
BurkinaFaso	100.00%	0
Dakar	91.67%	0
GuineaBissau	100.00%	0
Lunyo	100.00%	10% (A9)
Bundibugyo	91.67%	0
Kichwamba	100.00%	0
Kisimu	100.00%	10% (AG2)
**Percentage of missing data across dataset: 1.02%**		

Table S12: Microsatellite descriptive statistics per population.

Population	Region	Ho	uHe	Fis
Europa	SWIO	0.458	0.475	0.054
Ngari	West Africa	0.443	0.350	-0.211
Cameroon	West Africa	0.558	0.711	0.201
Gabon	West Africa	0.639	0.710	0.061
Burkina Faso	West Africa	0.617	0.683	0.067
Dakar	West Africa	0.600	0.522	-0.184
GuineaBissau	West Africa	0.617	0.671	0.087
Lunyo, Uganda	East Africa	0.675	0.591	-0.187
Bundibugyo, Uganda	East Africa	0.446	0.574	0.182
Kichwamba, Uganda	East Africa	0.542	0.624	0.099
Kenya	East Africa	0.646	0.716	0.080
Total		0.567	0.602	0.028

Ho: observed heterozygosity

uHe: unbiased expected heterozygosity

Fis: fixation index

West Africa Ho= 0.579

East Africa Ho= 0.577

Table S13: Per population, per locus results for violation of Hardy-Weinberg Equilibrium from microsatellite data

Population	# Loci out of HWE	Loci out of HWE
Europa	1	AG2
Ngari	3	AC4, A9, B3
Cameroon	1	B3
Gabon	1	A1
Burkina Faso	1	AG2
Dakar	0	
GuineaBissau	1	A1
Lunyo, Uganda	2	AG1, B3
Bundibugyo, Uganda	4	AC2, A1, B2, B3
Kichwamba, Uganda	0	
Kenya	0	

Table S14**.** Priors and posteriors (logistic approach) for the ABC analysis testing scenarios on the history of African *Aedes aegypti*

Parameter	Details	Prior	Posterior
Colonization scenarios:	**Scenario 1 –**	N/A	**A) 0.8864 [0.8475,0.9253]**
	**Europa *Ae. aegypti* to African *Ae. aegypti***		**B) 0.8481 [0.8165,0.8798]**
A) Gabon (WA), Uganda (EA), Europa			
	Scenario 2–		
B) Burkina Faso (WA), Kenya (EA), Europa	West African *Ae. aegypti* to Europa and East African *Ae. aegypti*	N/A	A) 0.0936 [0.0000,0.3949]
			B) 0.1158 [0.0000,0.2896]
	Scenario 3 –		
	East African *Ae. aegypti* to Europa and West African *Ae. aegypti*		A) 0.0200 [0.0000,0.0551]
		N/A	B) 0.0361 [0.0000,0.2366]
**SCENARIO 1**			
	Europa	100 – 500,000	A) 34,600 [19,700 – 55,300]
			B) 36,300 [18,800 – 57,700]
	West Africa	100 – 500,000	A) 378,000 [247,000 – 485,000]
Effective population size			B) 381,000 [255,000 – 485,000]
	East Africa	100 – 500,000	A) 233,000 [127,000 – 367,000]
			B) 324,000 [184,000 – 471,000]

Split time[1]	Africa from Europa	10,000 – 1,000,000	A) 353,000 [97,800 – 797,000]<br>B) 396,000 [119,000 – 835,000]
	West Africa from East Africa	10,000 – 1,000,000	A) 200,000 [78,100 – 372,000]<br>B) 249,000 [101,000 – 494,000]
Mutation rate	Microsatellite –Stepwise Mutation Model (SMM)	$9x10^{-06}$-$1x10^{-05}$	A) $9.47x10^{-06}$ [$9.05x10^{-06}$ - $9.94x10^{-06}$]<br>B) $9.46x10^{-06}$ [$9.04x10^{-06}$ - $9.94x10^{-06}$]
Confidence	Type I error<br>(Simulated under scenario 1)	N/A	A) 0.124<br>B) 0.306
	Type II error<br>(Simulated under scenario 2)	N/A	A) 0.054<br>B) 0.188
	Type II error<br>(Simulated under scenario 3)	N/A	A) 0.074<br>B) 0.066

[1] Time in generations (10 generations / year).

WA: West Africa

EA: East Africa

Table S15**:** Priors and posteriors (logistic approach) for the ABC analysis testing scenarios on the history of African *Aedes aegypti.* (Random sampled individuals from West Africa and East Africa pools).

**Parameter**	**Details**	**Prior**	**Posterior**
Colonization scenarios (3 independent replicas)	**Scenario 1** – **Europa *Ae. aegypti* to African *Ae. aegypti***	N/A	**A) 0.9199 [0.8967,0.9431]**<br>**B) 0.9561 [0.9379,0.9744]**<br>**C) 0.8764 [0.8359,0.9168]**
	Scenario 2– West African *Ae. aegypti* to Europa and East African *Ae. aegypti*	N/A	A) 0.0686 [0.0000,0.3338]<br>B) 0.0372 [0.0000,0.4333]<br>C) 0.1059 [0.0000,0.3903]
	Scenario 3 – East African *Ae. aegypti* to Europa and West African *Ae. aegypti*	N/A	A) 0.0115 [0.0000,0.3009]<br>B) 0.0067 [0.0000,0.4193]<br>C) 0.0177 [0.0000,0.3411]
**SCENARIO 1**			
Effective population size	Europa	100 – 500,000	A) 34,500 [19,900 – 54,300]<br>B) 29,200 [16,700 – 46,200]<br>C) 32,700 [18,700 – 51,800]

	West Africa	100 – 500,000	A) 333,000 [190,000 – 474,000]<br>B) 465,000 [412,000 – 497,000]<br>C) 364,000 [231,000 – 482,000]
	East Africa	100 – 500,000	A) 412,000 [313,000 – 489,000]<br>B) 414,000 [315,000 – 489,000]<br>C) 399,000 [293,000 – 485,000]
[1]Split time	Africa from Europa	10,000 – 1,000,000	A) 408,000 [149,000 – 843,000]<br>B) 340,000 [112,000 – 792,000]<br>C) 332,000 [106,000 – 781,000]
	West Africa from East Africa	10,000 – 1,000,000	A) 221,000 [90,300 – 441,000]<br>B) 171,000 [67,400 – 358,000]<br>C) 206,000 [82,800 – 471,000]
Mutation rate	Microsatellite –Stepwise Mutation Model (SMM)	$9x10^{-06}$-$1x10^{-05}$	A) $9.53x10^{-06}$ [$9.09x10^{-06}$ - $9.99x10^{-06}$]<br>B) $9.51x10^{-06}$ [$9.08x10^{-06}$ - $9.98x10^{-06}$]<br>C) $9.49x10^{-06}$ [$9.06x10^{-06}$ - $9.95x10^{-06}$]
Confidence	Type I error	N/A	A) 0.256

(Simulated under scenario 1)		B) 0.260
		C) 0.260
Type II error		A) 0.230
(Simulated under scenario 2)	N/A	B) 0.220
		C) 0.168
Type II error		A) 0.086
(Simulated under scenario 3)		B) 0.096
	N/A	C) 0.096

[1] Time in generations (10 generations / year).

Table S16: Calibration information used in BEAST analyses

**Fossil For Node Calibration**	Minimum age	Maximum age	Explanation
Aedes (Ocherlotatus) serafini Szadziewski 1998	33.9	123.87	This fossil is an Eocene Baltic amber inclusion and was placed in *Ochlerotatus*, currently a subgenus of *Aedes*, by Szadziewski & Gilka, 2011. The uniform calibration corresponding to this fossil was placed at the common ancestor of *Aedes* mosquitoes in this analysis. The maximum age follows the maximum age of the common ancestor of *Aedes* in Reidenbach et al. 2009.
Culiseta kishenehn Harbach & Greenwalt, 2012	46.2	218.81	This compression fossil from the Kishenehn shale deposits in Montana was placed in an extinct subgenus within *Culiseta* by Harbach and Greenwalt, 2012. The uniform calibration corresponding to this fossil was placed at the common ancestor of *Culiseta* and the tribe Aedini. The maximum age follows the maximum age of the common ancestor of these two clades in Reidenbach et al. 2009.
Culex (Culex) erikae Szadziewski & Szadziewska, 1985	33.9	218.81	This fossil is an Eocene Baltic amber inclusion placed in the subgenus *Culex* for its close affinity to *Culex (Culex) pipiens*. The uniform calibration corresponding to this fossil was placed at the common ancestor of *Culex (Culex) quinquefasciatus* – a close relative of *Culex pipiens* – and *Culex (Neoculex) territans*. The maximum age constraint follows the maximum age of the common ancestor of the genus *Culex* and its nearest relative in Reidenbach et al. 2009
Paleoculicis minutus Poinar et al 2000	76.5	226.22	This fossil male mosquito was reported by Poinar et al. 2000. The specimen was found in Canadian cretacious amber in a formation ranging in age from 76.5 to 79.5 MYA. The aforementioned authors placed the specimen as in the Culicinae, but its affinities with tribes within the family is unknown (see Poinar et al. 2000 for a discussion). The maximum age is based off of Reidenbach et al. 2009.

Table S17: The analysis of variance comparing fraction of missing SNPs with population of origin for each sample.

	Df	Sum of Squares	Mean Squares	F-statistic	P-value
**Population**	18	0.54	0.03	122.2	<2e-16
**Residual**	180	0.04	0.00		
